# Association of Cervical Disease and Metabolic Comorbidities with Adhesive Capsulitis in Patients with Shoulder Pain: A Multivariate Analysis

**DOI:** 10.3390/medicina62061144

**Published:** 2026-06-11

**Authors:** Chang-Hyung Lee, Siwon Yoon, Jung Hyun Yang, Min-Hyeok Choi, Min Hui Moon, Kyeong-Baek Kim, Suk Woong Kang

**Affiliations:** 1 Department of Rehabilitation Medicine, Research Institute for Convergence of Biomedical Science and Technology, Pusan National University Yangsan Hospital, School of Medicine, Pusan National University, Yangsan 626-770, Republic of Korea; aarondoctor@gmail.com; 2 Pukyong National University Industry—University Cooperation Foundation, Busan 48513, Republic of Korea; aa01066863594@gmail.com; 3 Department of Rehabilitation Medicine, Pusan National University Yangsan Hospital, Pusan National University, Yangsan 626-770, Republic of Korea; dr.yjh314@gmail.com; 4 Department of Preventive and Occupational & Environmental Medicine, Research Institute for Convergence of Biomedical Science and Technology, Pusan National University Yangsan Hospital, School of Medicine, Pusan National University, Yangsan 626-770, Republic of Korea; come2mh@gmail.com (M.-H.C.); pnuyh3255@gmail.com (M.H.M.); 5 Office of Public Health Service, Pusan National University Yangsan Hospital, School of Medicine, Pusan National University, Yangsan 626-770, Republic of Korea; 6 Department of Orthopedics, Research Institute for Convergence of Biomedical Science and Technology, Pusan National University Yangsan Hospital, School of Medicine, Pusan National University, Yangsan 626-770, Republic of Korea; pnuyh.102@gmail.com

**Keywords:** shoulder, cervical disease, adhesive capsulitis, etiology

## Abstract

Background: The prevalence of adhesive capsulitis (AC) is estimated to be 2–5% in the general population. However, the etiology of AC remains unclear. Among the various proposed factors, the precise role of cervical disease, and the severity of cervical degeneration, in the development of AC has not been fully elucidated. This study aimed to analyze the contribution of cervical disease to AC in patients with shoulder pain. Methods: A total of 409 patients who visited the Department of Rehabilitation Medicine for shoulder pain were retrospectively reviewed. The outcome variable was the presence of AC. In addition to cervical disease, other independent variables affecting AC, including sex, diabetes, obesity, dyslipidemia, thyroid disease, immobilization after surgery, rotator cuff tear, subacromial spur, and shoulder joint osteoarthritis were reviewed. To compare the two groups, an independent *t*-test or chi-square test was performed for continuous and categorical data. Multivariate regression analysis was used to assess the effects of independent factors on AC, adjusting for confounders. Results: Among the 409 patients, 176 (43.0%) were diagnosed with AC. Multivariate analysis demonstrated that diabetes (OR 3.03, 95% CI 1.55–5.91, *p* = 0.001) and cervical disease (OR 3.03, 95% CI 1.75–5.25, *p* < 0.001) were significantly associated with increased odds of AC. In contrast, increasing age (OR 0.95, 95% CI 0.92–0.98, *p* = 0.007), dyslipidemia (OR 0.55, 95% CI 0.31–0.98, *p* = 0.044), and postoperative immobilization (OR 0.64, 95% CI 0.41–0.99, *p* = 0.046) were associated with decreased odds of AC. The prevalence of AC increased with the severity of cervical degeneration. Conclusion: In patients with shoulder pain, diabetes and cervical disease were positively associated with AC, whereas age, dyslipidemia, and postoperative immobilization showed inverse associations. These findings suggest that both metabolic and cervical factors may contribute to the development of AC, highlighting the importance of considering cervical pathology in patients with shoulder pain.

## 1. Introduction

Adhesive capsulitis (AC), also known as “frozen shoulder”, is a common condition characterized by contracture of the glenohumeral capsule [1,2,3]. Clinically, it presents with pain, stiffness, and dysfunction of the affected shoulders [4,5,6]. The prevalence of AC is estimated to be 2–5% in the general population. Most patients diagnosed with adhesive capsulitis are women aged 40–60 years old [7,8]. Although AC is often considered a self-limiting condition, symptoms may persist for several years, and some patients never fully regain normal shoulder function [9].

Beyond its clinical burden, AC also imposes a significant economic impact on the healthcare system. Previous studies have reported that the average treatment cost for patients undergoing physical therapy may reach approximately $6150, with wide variability depending on treatment duration and interventions [10]. More invasive procedures, such as manipulation under anesthesia, are associated with substantially higher procedural costs, creating an additional financial burden for both patients and healthcare providers [11,12].

Despite its relatively high prevalence, the exact pathophysiology of AC remains incompletely understood. Traditionally, AC has been classified into primary (idiopathic) and secondary forms. Primary AC develops without an identifiable underlying cause, whereas secondary AC occurs in association with systemic, intrinsic, or extrinsic factors affecting the shoulder joint [13,14].

Previous studies have identified several systemic and local factors associated with AC, including diabetes mellitus, thyroid disease, dyslipidemia, obesity, postoperative immobilization, and rotator cuff pathology [8,9,12,15,16,17]. These factors are believed to contribute to capsular inflammation, fibrosis, and altered shoulder biomechanics, ultimately leading to joint stiffness.

Among the potential etiological factors, cervical spine pathology has received increasing attention as a possible contributor to AC development. Cervical radiculopathy, particularly involving the C5 and C6 nerve roots, may affect the muscles responsible for stabilizing and coordinating glenohumeral joint motion [16,18,19,20,21,22,23,24]. Chronic neural irritation can lead to weakness in the innervated shoulder girdle muscles, resulting in altered muscle activation patterns and imbalance of the rotator cuff and scapular stabilizers. Such neuromuscular dysfunction may contribute to abnormal shoulder mechanics and eventually promote capsular contracture.

Furthermore, cervical spine disorders may produce radiating pain, sensory disturbances, and reflex changes that overlap with symptoms of shoulder pathology. This clinical overlap may lead to delayed diagnosis or coexistence of cervical and shoulder disorders in the same patient. Several studies have suggested that cervical radiculopathy and cervical spondylosis may be associated with an increased risk of developing AC [16,19,20,21,22,23,24]. However, the precise contribution of cervical disease to AC development, particularly in relation to other established metabolic and shoulder-related risk factors, remains incompletely understood.

In addition, the potential influence of cervical degenerative changes on the occurrence of AC has not been sufficiently investigated. While cervical radiculopathy may reflect active neural irritation, radiologic cervical degeneration may represent chronic structural changes that could alter neuromuscular control of the shoulder girdle [16].

Therefore, the purpose of this study was to investigate, using multivariate analysis, the factors associated with AC in patients presenting with shoulder pain, to explore whether these associations differed according to sex and age, and to evaluate the association between the severity of cervical degenerative changes and AC prevalence.

## 2. Materials and Methods

This study was approved by the Institutional Review Board of Pusan National University Yangsan Hospital (IRB No.05-2025-048).

### 2.1. Study Population

This retrospective study included patients diagnosed with shoulder pain between January 2021 and December 2021. A total of 886 patients who visited the Department of Rehabilitation Medicine for shoulder pain during the study period were analyzed. Plain radiographs of the shoulder and neck were obtained for all patients.

In this study, the exclusion criteria were as follows:

(1) Patients with upper-extremity dysfunction due to brain damage
(2) Patients with previous history of shoulder surgery

After applying these exclusion criteria, a total of 409 patients with shoulder pain were included in the final analysis. All cases were retrospectively reviewed by a specialist in rehabilitation medicine and an orthopedic surgeon, each with more than 15 years of clinical experience.

### 2.2. Variables

The outcome variable was the presence of adhesive capsulitis (AC) diagnosed through physical examination. AC was defined as global reduction in the range of motion with equal passive and active ranges of motion (flexion < 100°, external rotation < 10°, and internal rotation < L5) [13].

In addition to gender and age, the presence of obesity (BMI ≥ 25 kg/m^2^), diabetes (fasting blood sugar level ≥ 126 mg/dL or use of diabetes medication), thyroid disease, dyslipidemia and immobilization after surgery were selected as independent variables. Immobilization after surgery was defined as immobilization for at least 1 month following surgeries other than shoulder surgery, such as thoracic or breast surgery.

The presence of subacromial spurs and osteoarthritis was assessed using plain radiography. Rotator cuff tears were evaluated using ultrasonography or shoulder MRI.

The presence and severity of cervical disease were also included as independent variables and were defined based on radiographic findings and neurological symptoms. Cervical disease was assessed based on clinical symptoms, including radiating pain, numbness, tingling sensations, muscle weakness, and diminished reflexes.

The severity of cervical disease was defined radiologically as intervertebral disc height narrowing at the C4/5 or C5/6 level according to the Pfirrmann classification [19], categorized as normal (group 1), slightly decreased (group 2), or moderately to severely decreased (group 3). The presence of cervical radiculopathy confirmed by electrodiagnostic studies was also classified as group 3.

### 2.3. Statistics

Patients were divided into two groups according to the presence or absence of adhesive capsulitis (AC). To compare the two groups, independent *t*-tests and chi-square tests were performed for continuous and categorical variables, respectively. Multivariate logistic regression analysis was conducted to identify factors associated with AC in patients with shoulder pain. In addition, subgroup analyses stratified by sex and age (<60 years and ≥60 years) were performed using multivariate logistic regression models to explore whether factors associated with AC differed across these subgroups. Odds ratios (ORs) with 95% confidence intervals (CIs) were calculated for each variable in the multivariate models. All statistical analyses were performed using Stata/MP software, version 17.0 (Stata Corporation, College Station, TX, USA). A *p*-value < 0.05 was considered statistically significant.

## 3. Results

Table 1 presents the general characteristics of the study participants. Among the 409 patients with shoulder pain, 222 (54.3%) were female, and the mean age was 56 ± 6.7 years. Adhesive capsulitis (AC) was diagnosed in 176 patients (43.0%). The prevalence rates of diabetes, dyslipidemia, and thyroid disease were 14.4%, 18.8%, and 6.8%, respectively. Immobilization after surgery, rotator cuff tears, and cervical disease were observed in 42.1%, 33.2%, and 19.8% of patients, respectively.

### 3.1. Comparison of Independent Variables According to Adhesive Capsulitis (AC)

Table 2 presents the results of the univariate analysis according to the presence of AC. Significant differences were observed for age, diabetes, postoperative immobilization, and cervical disease (*p* < 0.05). The mean age of patients with AC was 54.8 ± 6.0 years, which was significantly lower than that of patients without AC (57.0 ± 6.4 years, *p* < 0.001). The prevalence of AC was significantly higher in patients with diabetes than in those without diabetes (64.4% vs. 39.4%, *p* < 0.001). Likewise, AC was more common in patients with cervical disease than in those without cervical disease (66.7% vs. 37.2%, *p* < 0.001). Regarding postoperative immobilization, the prevalence of AC was significantly higher in patients who did not undergo immobilization than in those who did (47.3% vs. 37.2%, *p* = 0.043).

### 3.2. Multivariate Logistic Regression Analysis on the Influence of Factors on the Development of Adhesive Capsulitis (AC)

Table 3 presents the results of the multivariate logistic regression analysis identifying factors independently associated with AC in patients with shoulder pain. Among the variables examined, diabetes (OR 3.03, 95% CI 1.55–5.91, *p* = 0.001) and cervical disease (OR 3.03, 95% CI 1.75–5.25, *p* < 0.001) were significantly associated with increased odds of AC, indicating that both metabolic and cervical factors may contribute to its development. In contrast, increasing age was associated with reduced odds of AC (OR 0.95, 95% CI 0.92–0.98, *p* = 0.007), suggesting that younger patients with shoulder pain may be more susceptible to AC in this cohort. Dyslipidemia (OR 0.55, 95% CI 0.31–0.98, *p* = 0.044) and postoperative immobilization (OR 0.64, 95% CI 0.41–0.99, *p* = 0.046) were also inversely associated with AC. No significant associations were observed for sex, obesity, thyroid disease, rotator cuff tear, subacromial spur, or osteoarthritis (all *p* > 0.05).

### 3.3. Influence of Etiologic Factors on the Development of Adhesive Capsulitis (AC) According to Sex

Table 4 presents the sex-specific associations between clinical factors and AC. Among all the patients, 222 (54.3%) were female. In male patients, age, diabetes, dyslipidemia, and cervical disease were significantly associated with AC. In female patients, diabetes, obesity and cervical disease were significantly associated with AC. Notably, diabetes and cervical disease were consistently associated with AC development in both sexes.

### 3.4. Influence of Etiologic Factors on the Development of Adhesive Capsulitis (AC) According to Age

Table 5 presents the influence of clinical factors on AC according to age group. In patients younger than 60 years old, diabetes, dyslipidemia, postoperative immobilization, subacromial spurs, and cervical disease were significantly associated with AC. Among these factors, diabetes showed the strongest association with AC (OR 12.18, 95% CI 3.78–39.26, *p* < 0.001). In patients aged 60 years old or older, only cervical disease remained significantly associated with AC in the multivariate analysis (OR 4.53, 95% CI 1.70–12.09, *p* = 0.003).

### 3.5. Influence of Etiologic Factors on the Development of Adhesive Capsulitis (AC) According to the Severity of Cervical Disease

Table 6 presents the relationship between the severity of cervical degeneration and the prevalence of adhesive capsulitis. The prevalence of AC increased progressively with the severity of cervical degeneration. The prevalence rates of AC were 33.3% in Group 1 (no radiographic degeneration), 51.3% in Group 2 (mild degeneration), and 63.5% in Group 3 (moderate-to-severe degeneration). A chi-square analysis demonstrated a significant difference in AC prevalence among the three groups (*p* < 0.001). These findings suggest that the risk of adhesive capsulitis increases with the severity of cervical degenerative changes.

## 4. Discussion

In this study, cervical disease and diabetes were positively associated with adhesive capsulitis (AC), whereas increasing age, dyslipidemia, and postoperative immobilization showed inverse associations. These findings suggest that both metabolic and cervical factors may contribute to the development of AC, while some clinical variables may exhibit inverse relationships within this patient population.

The relationship between cervical disease and AC may be explained by the role of the C5 and C6 nerve roots, which innervate the shoulder girdle and rotator cuff muscles. Cervical radiculopathy can lead to muscle weakness, imbalance, and altered activation patterns, resulting in abnormal shoulder biomechanics. Previous studies have reported associations between AC and cervical radiculopathy or cervical spondylosis [16,19,20,21,22,23,24], and overlapping symptoms between cervical and shoulder disorders may complicate diagnosis [22,23]. In addition, altered neuromuscular control and nerve-related dysfunction have been suggested as contributing mechanisms linking cervical pathology to AC [25,26,27].

Furthermore, the prevalence of AC increased with the severity of cervical degeneration, suggesting that structural changes in the cervical spine may contribute to shoulder dysfunction. In addition, cervical disease showed a strong independent association with AC in the multivariate analysis, indicating that both radiologic degeneration and clinically significant cervical pathology may play important roles in the development of AC.

Diabetes was also significantly associated with AC in our study, consistent with previous reports [14,16,19]. Chronic hyperglycemia may promote capsular fibrosis and inflammatory changes and increase susceptibility to peripheral nerve dysfunction, potentially amplifying the effects of cervical pathology.

In contrast, dyslipidemia showed an inverse association with AC in our analysis. However, previous studies have reported inconsistent findings, and some have suggested that the association between dyslipidemia and AC may be more pronounced in patients with coexisting metabolic conditions such as diabetes [26,27,28]. This implies that dyslipidemia alone may not be sufficient to increase the risk of AC, but may contribute in combination with other metabolic abnormalities. Therefore, the inverse association observed in our study may reflect differences in patient characteristics or unmeasured metabolic factors, and should be interpreted with caution.

Interestingly, patients with AC were younger than those without AC in the present study. This finding may be partly explained by the known epidemiologic pattern of AC, which most commonly affects individuals in the fifth to sixth decades of life, with peak incidence reported around the mid-50s [26]. In older patients with shoulder pain, age-related shoulder disorders such as rotator cuff disease and osteoarthritis become increasingly prevalent, which may alter the relative distribution of shoulder diagnoses within this population [29,30]. Therefore, the inverse association between age and AC observed in our cohort should be interpreted cautiously and may reflect differences in the underlying spectrum of shoulder disorders rather than a direct protective effect of older age.

The subgroup analyses further demonstrated that cervical disease remained significantly associated with adhesive capsulitis across both sex and age categories. In contrast, the associations of several metabolic factors differed according to patient characteristics. Diabetes was consistently associated with AC in both men and women, whereas obesity showed a significant association only among women. Similarly, diabetes was strongly associated with AC in patients younger than 60 years, while cervical disease remained the only significant factor in patients aged 60 years or older. These findings suggest that although metabolic risk factors may vary across demographic subgroups, the association between cervical pathology and AC appears to be relatively consistent, supporting its potential role as an important contributor to AC development [16,29].

This study has several limitations. First, due to its retrospective design, the temporal relationship between cervical pathology and the development of AC could not be determined. Sequential changes in potential etiological factors were also not evaluated. Prospective studies are therefore required to clarify the causal relationship among these variables. Second, cervical radiculopathy was not systematically assessed in all patients. MRI and electromyography were performed only in patients with severe cervical symptoms, which may have limited the ability to fully evaluate neural involvement. Third, although the prevalence of AC increased with the severity of cervical degeneration, the severity analysis was exploratory in nature. In this study, cervical disease and cervical degeneration severity represented distinct clinical and radiographic constructs. Cervical disease was defined according to neurological symptoms and clinical findings, whereas cervical degeneration severity was based on radiographic assessment. Therefore, the severity findings should be interpreted with caution, and further studies are needed to determine the independent contributions of clinical cervical pathology and radiographic degeneration to the development of AC. Future prospective studies incorporating comprehensive cervical evaluations may improve the generalizability of these findings and further clarify the relationship between cervical degeneration severity and adhesive capsulitis.

## 5. Conclusions

In patients presenting with shoulder pain, diabetes and cervical disease were positively associated with adhesive capsulitis (AC), whereas increasing age, dyslipidemia, and postoperative immobilization showed inverse associations. In addition, the prevalence of AC increased with greater severity of cervical degenerative changes.

These findings suggest that both metabolic factors and cervical pathology may contribute to the development of AC. Notably, cervical disease was independently associated with AC, and the prevalence of AC increased according to the severity of cervical degeneration. These results highlight the importance of considering both clinical cervical pathology and radiographic cervical degeneration when evaluating patients with shoulder pain. Further prospective studies are warranted to clarify the temporal relationship and underlying mechanisms linking cervical pathology and AC.

## Figures and Tables

**Table 1:** General characteristics of the participants.

		N	%
Sex	Female	222	54.3
	Male	187	45.7
Age (years, Mean ± SD, range)		56 ± 6.7, 40–65	
AC	(−)	233	57.0
	(+)	176	43.0
Diabetes	(−)	350	85.6
	(+)	59	14.4
Obesity	(−)	273	66.8
	(+)	136	33.2
Dyslipidemia	(−)	332	81.2
	(+)	77	18.8
Thyroid disease	(−)	381	93.2
	(+)	28	6.8
Immobilization after surgery	(−)	237	57.9
	(+)	172	42.1
Rotator cuff tear	(−)	273	66.8
	(+)	136	33.2
Subacromial spur	(−)	162	39.6
	(+)	247	60.4
Osteoarthritis	(−)	342	83.6
	(+)	67	16.4
Cervical disease	(−)	328	80.2
	(+)	81	19.8

“(−) indicates the absence of the condition, and (+) indicates the presence of the condition.”

**Table 2:** Comparison of independent variables according to adhesive capsulitis (AC).

		AC (−)		AC (+)		
		N	%	N	%	*p*
Sex	Female	120	51.50	102	57.95	0.195
	Male	113	48.50	74	42.05	
Age (Mean ± SD)		57.0 ± 6.4		54.8 ± 6.0		<0.001 *
Diabetes	(−)	212	90.99	138	78.41	<0.001 *
	(+)	21	9.01	38	21.59	
Obesity	(−)	148	63.52	125	71.02	0.111
	(+)	85	36.48	51	28.98	
Dyslipidemia	(−)	182	78.11	150	85.23	0.068
	(+)	51	21.89	26	14.77	
Thyroid disease	(−)	219	93.99	162	92.05	0.440
	(+)	14	6.01	14	7.95	
Immobilization after surgery	(−)	125	53.65	112	63.64	0.043 *
	(+)	108	46.35	64	36.36	
Rotator cuff tear	(−)	149	63.95	124	70.45	0.167
	(+)	84	36.05	52	29.55	
Subaromial spur	(−)	99	42.49	63	35.80	0.171
	(+)	134	57.51	113	64.20	
Osteoarthritis	(−)	193	82.83	149	84.66	0.621
	(+)	40	17.17	27	15.34	
Cervical disease	(−)	206	88.41	122	69.32	<0.001 *
	(+)	27	11.59	54	30.68	

*: *p*-values < 0.05, considered statistically significant. (−) indicates the absence of the condition, and (+) indicates the presence of the condition.

**Table 3:** Results of multivariate logistic regression analysis on the influence of factors on adhesive capsulitis (AC).

		OR (95% CI) ^1^	*p*
Sex	Male	Reference	
	Female	0.78 (0.50–1.21)	0.281
Age		0.95 (0.92–0.98)	0.007 *
Diabetes	(−)	Reference	
	(+)	3.03 (1.55–5.91)	0.001 *
Obesity	(−)	Reference	
	(+)	0.62 (0.39–1.00)	0.054
Dyslipidemia	(−)	Reference	
	(+)	0.55 (0.31–0.98)	0.044 *
Thyroid disease	(−)	Reference	
	(+)	0.97 (0.39–2.39)	0.950
Immobilization after surgery	(−)	Reference	
	(+)	0.64 (0.41–0.99)	0.046 *
Rotator cuff tear	(−)	Reference	
	(+)	0.72 (0.45–1.14)	0.169
Subacromial spur	(−)	Reference	
	(+)	1.48 (0.94–2.33)	0.086
Osteoarthritis	(−)	Reference	
	(+)	0.86 (0.47–1.58)	0.648
Cervical disease	(−)	Reference	
	(+)	3.03 (1.75–5.25)	<0.001 *

^1^ OR (95% CI): odds ratio (95% confidence interval); *: *p*-values < 0.05, considered statistically significant. (−) indicates the absence of the condition, and (+) indicates the presence of the condition.

**Table 4:** Influence of factors on adhesive capsulitis (AC) according to gender.

		Male		Female	
		OR (95% CI) ^1^	*p*	OR (95% CI)	*p*
Age		0.94 (0.89 –0.98)	0.016 *	0.97 (0.92–1.01)	0.184
Diabetes	(−)	Reference		Reference	
	(+)	4.38 (1.59–12.08)	0.004 *	3.23 (1.18–8.85)	0.022 *
Obesity	(−)	Reference		Reference	
	(+)	1.00 (0.51–1.95)	0.995	0.35 (0.17–0.74)	0.006 *
Dyslipidemia	(−)	Reference		Reference	
	(+)	0.36 (0.14–0.92)	0.033 *	0.61 (0.28–1.32)	0.217
Thyroid disease	(−)	Reference		Reference	
	(+)	0.25 (0.27–2.44)	0.238	1.17 (0.41–3.34)	0.766
Immobilization after surgery	(−)	Reference		Reference	
	(+)	0.79 (0.40–1.55)	0.506	0.63 (0.35–1.15)	0.139
Rotator cuff tear	(−)	Reference		Reference	
	(+)	0.56 (0.27–1.18)	0.129	0.77 (0.41–1.45)	0.429
Subacromial spur	(−)	Reference		Reference	
	(+)	1.84 (0.89–3.79)	0.096	1.41 (0.76–2.62)	0.266
Osteoarthritis	(−)	Reference		Reference	
	(+)	1.91 (0.76–4.79)	0.165	0.47 (0.19–1.11)	0.088
Cervical disease	(−)	Reference		Reference	
	(+)	3.86 (1.60–9.31)	0.003 *	2.54 (1.22–5.29)	0.012 *

^1^ OR (95% CI): odds ratio (95% confidence interval); *: *p*-values < 0.05, considered statistically significant; (−) indicates the absence of the condition, and (+) indicates the presence of the condition.

**Table 5:** Influence of etiologic factors on the development of adhesive capsulitis (AC) according to age.

		˂60		≥60	
		OR (95% CI) ^1^	*P*	OR (95% CI)	*p*
Sex		0.85 (0.46–1.55)	0.607	0.79 (0.38–1.61)	0.522
Diabetes	(−)	Reference		Reference	
	(+)	12.18 **^+^** (3.78–39.26)	<0.001 *	0.46 (0.15–1.45)	0.189
Obesity	(−)	Reference		Reference	
	(+)	0.58 (0.31–1.10)	0.097	0.56 (0.25–1.21)	0.142
Dyslipidemia	(−)	Reference		Reference	
	(+)	0.40 (0.17–0.95)	0.038	0.80 (0.35–1.81)	0.607
Thyroid disease	(−)	Reference		Reference	
	(+)	0.68 (0.17–2.68)	0.587	0.99 (0.24–4.05)	0.995
Immobilization after surgery	(−)	Reference		Reference	
	(+)	0.48 (0.26–0.87)	0.016	1.26 (0.61–2.63)	0.524
Rotator cuff tear	(−)	Reference		Reference	
	(+)	0.65 (0.34–1.24)	0.195	0.60 (0.29–1.25)	0.177
Subacromial spur	(−)	Reference		Reference	
	(+)	1.88 (1.01–3.50)	0.046	1.15 (0.57–2.33)	0.683
O steoarthritis	(−)	Reference		Reference	
	(+)	0.84 (0.36–1.94)	0.693	0.71 (0.27–1.84)	0.489
Cervical disease	(−)	Reference		Reference	
	(+)	2.97 (1.44–6.12)	0.003 *	4.53 (1.70–12.09)**^+^**	0.003 *

*: *p*-values < 0.05, considered statistically significant. **^+^**: Highest odds ratio for each group; (−) indicates the absence of the condition, and (+) indicates the presence of the condition.

**Table 6:** Influence of etiologic factors on the development of adhesive capsulitis (AC) according to the severity of cervical disease.

	Group 1 (n = 246)	Group 2 (n = 78)	Group 3 (n = 85)	*p* Value
AC (−)	164	38	31	0.000
AC (+)	82 (33.3%)	40 (51.3%)	54 (63.5%)	

Group 1: absence of C4/5 or C5/6 intervertebral height of narrowing in plain sagittal radiographs; Group 2: slightly decreased intervertebral disc height; Group 3: Moderately to severely decreased intervertebral disc height according to the Pfirmann classification [19]. The presence of cervical radiculopathy confirmed by electrodiagnostic studies was also classified into Group 3. (−) indicates the absence of the condition, and (+) indicates the presence of the condition.

## Data Availability

The data presented in this study are not publicly available due to privacy and ethical restrictions but are available from the corresponding author upon reasonable request.
